# Enoki mushroom residue-based biochar loaded with *Bacillus megaterium* YZS-M06 improves coastal sandy soil and promotes *Ipomoea pes-caprae* growth

**DOI:** 10.1186/s40643-026-01073-w

**Published:** 2026-05-26

**Authors:** Zongsheng Yuan, Jie Yang, Sifan Wang, Xun Dong, Xiaoling Wang, Fang Liu

**Affiliations:** 1 Fujian Key Laboratory on Conservation and Sustainable Utilization of Marine Biodiversity, Fuzhou Institute of Oceanography, College of Geography and Oceanography, Minjiang University, Fuzhou, 350108 Fujian China; 2 College of Environment and Safety Engineering, Fuzhou University, Fuzhou, 350108 Fujian China; 3 College of Life Sciences, Fujian Agriculture and Forestry University, Fuzhou, 350002 Fujian China; 4 College of Resources and Environment, Fujian Agriculture and Forestry University, Fuzhou, 350002 Fujian China

**Keywords:** *Bacillus megaterium*, Coastal sandy land, *Flammulina velutipes* residue-based biochar, Soil micro-ecology, Soil amelioration

## Abstract

In this study, biochar was prepared from *Flammulina velutipes* residues, and then loaded with *Bacillus megaterium* YZS-M06, to construct a composite material (BCB treatment) with synergistic remediation function. The improvement effect of this material on coastal sandy soil and its regulatory mechanism on the growth of *Ipomoea pes-caprae* were investigated. The findings demonstrated that *F. velutipes* residue-based biochar loaded with *B. megaterium* YZS-M06 (BCB) exerted a pronounced synergistic effect on the amelioration of chemical properties in coastal sandy land. After 30 days of cultivation, BCB treatment significantly increased soil total nitrogen (0.65 g/kg), total potassium (12.89 g/kg), and available potassium (81.75 mg/kg), as well as urease and phosphatase activities by 221% and 210% relative to the control. It also optimized the soil bacterial community, markedly enriching Bacillus from 46.58% to 57.85% relative abundance, reshaping microbial networks, and restructuring key ecological diversity–function pathways. These combined improvements in soil physicochemical properties and microbial structure effectively promoted *I. pes-caprae* growth. BCB treatment enhanced root length by 101% and root number by 63%, activated leaf antioxidant systems, mitigated membrane lipid peroxidation, and boosted osmolyte accumulation, thereby systematically strengthening the plant’s stress resistance. *F. velutipes* residue-based biochar loaded with *B. megaterium* YZS-M06, through the synergistic interaction of “biochar-functional bacteria”, substantially improved soil chemical properties, optimized microbial community architecture, enhanced stress tolerance of *I. pes-caprae*, and promoted plant growth. This study combines the resource utilization of agricultural waste *F. velutipes* residues with functional microbial enhancement. Through the synergistic mechanism of biochar-functional bacteria, the dual goals of coastal sandy soil improvement and plant growth promotion were achieved, providing a new technical route and theoretical basis for ecological restoration in extreme habitats.

**Graphical abstract:**

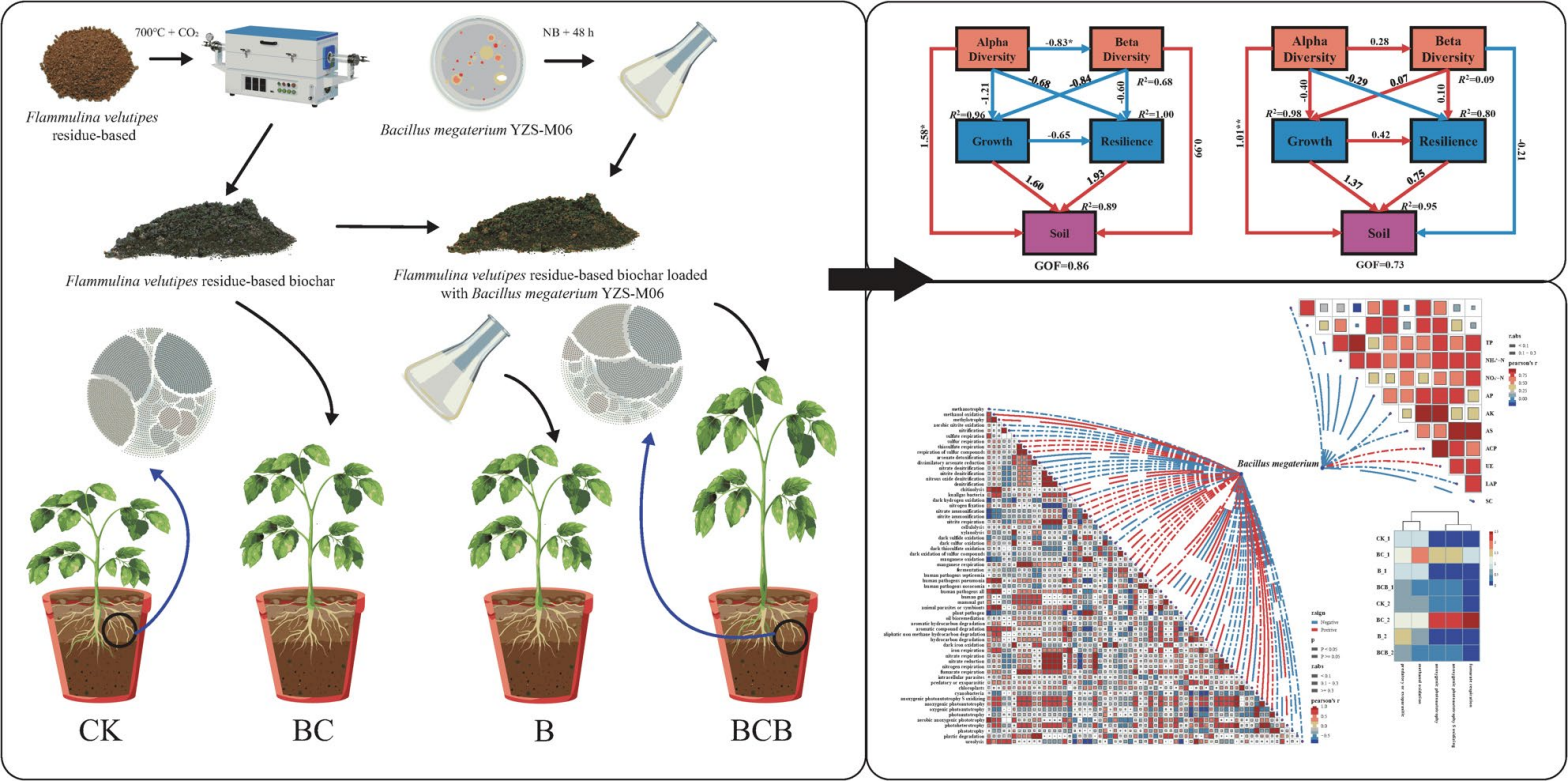

**Supplementary Information:**

The online version contains supplementary material available at 10.1186/s40643-026-01073-w.

## Introduction

Coastal sandy land, as a distinctive ecosystem shaped by the interaction between marine and terrestrial processes, not only functions as a natural protective barrier against storm surges and wind erosion but also plays a pivotal role in sustaining biodiversity within coastal zones (Douglas et al. 2025). Nevertheless, under the combined pressures of global climate change and intensified human activities, this ecosystem has been increasingly threatened by severe ecological challenges, including soil desertification, aggravated salinization, and progressive nutrient depletion (Élise et al. 2023). Among the currently available soil amelioration approaches for coastal sandy land, physical methods are characterized by high economic costs and limited long-term sustainability, chemical amelioration is prone to inducing secondary environmental pollution, and biological strategies are constrained by short microbial persistence and inadequate functional stability (Sun et al. 2025).

Biochar is a carbon-rich material produced through the thermal degradation of biomass, including agricultural and forestry wastes, under oxygen-limited conditions (Hakimi et al. 2026; Navas-Romero et al. 2026). Owing to its stable aromatic framework and highly developed porous architecture, carbon sequestration is facilitated, while heavy metals and organic pollutants in the environment are effectively adsorbed or immobilized (Li et al. 2025; Wang et al. 2026). Due to its distinctive physicochemical properties, biochar has been widely recognized as a promising material for soil amelioration. Biochar provides a new approach for soil improvement due to its unique physical and chemical properties. The feedstock of biochar determines its core properties and application functions, forming a trade-off between stable carbon sequestration and rapid nutrient supply (Xia et al. 2024). Lignocellulose-based biochar exhibits excellent chemical stability and long-term carbon sequestration potential, owing to its high carbon content and low atomic H/C and O/C ratios.

Additionally, the well-developed pore structure and large specific surface area impart favorable soil water retention capacity and enhanced pollutant adsorption performance (Enders et al. 2012). In recent years, the global edible fungus industry has experienced rapid expansion. In China, for example, total edible fungus production reached 44.37 million tons in 2024, rendering the disposal of spent mushroom substrate an increasingly prominent challenge (Atallah et al. 2021). Spent mushroom substrate is primarily composed of lignocellulose, abundant fungal proteins, microbial metabolites, and incompletely utilized nutrients. Biochar derived from spent mushroom substrate has been demonstrated to function as an effective soil amendment (Deng et al. 2020). However, its standalone application has been shown to be insufficient for sustaining long-term restoration of soil micro-ecology (Cui et al. 2021; Zhang et al. 2022). Moreover, microorganisms play a key role in nutrient transformation and stress alleviation (Soliman et al. 2025). In terms of enhancing nutrient cycling, they secrete extracellular enzymes to decompose organic matter and synthesize humus, thereby releasing nutrients and improving soil structure. For example, phosphate-solubilizing and potassium-solubilizing bacteria secrete organic acids or phosphatases to activate insoluble phosphorus and potassium minerals in soil into plant-available forms (Massucato et al. 2025).In terms of improving crop salt tolerance, they maintain osmotic balance by synthesizing compatible solutes and upregulating the expression of related genes in plants, while expressing Na⁺/H⁺ antiporters to exclude excessive sodium ions and maintain cellular ion homeostasis (Dindhoria et al. 2025).In terms of comprehensively promoting plant growth and health, rhizosphere growth-promoting bacteria and endophytes can synthesize and regulate plant hormones (e.g., auxin IAA), directly stimulating root development to enhance nutrient uptake (Dindhoria et al. 2025). Therefore, increasing attention has been paid to the synergistic potential between biochar and microorganisms. The unique porous structure of biochar enhances soil aeration and provides nutrient resources and stable microhabitats for microbial proliferation, thereby protecting microorganisms from adverse environmental conditions, including drought, unfavorable pH levels, and high salinity. Moreover, nutrient availability to microorganisms can be improved by biochar amendment, soil environmental conditions can be optimized, and the colonization of beneficial bacteria can be promoted (Deng et al. 2023). Collectively, these findings support the superior effectiveness of integrated “biochar-functional bacteria” strategies for soil amelioration.

Although numerous studies have investigated the roles of biochar or functional microorganisms in soil improvement, systematic research is still lacking on the synergistic remediation strategy using edible mushroom residue-derived biochar as a carrier for immobilizing specific functional strains, especially in the special habitat of coastal sandy land. On this basis, this study examined the ameliorative effects of *Flammulina velutipes* residue-based biochar (FV-biochar) loaded with *Bacillus megaterium* YZS-M06 on coastal sandy land soil. The following hypotheses were proposed: (1) At the soil nutrient level, *F. velutipes* residue-based biochar loaded with *B. megaterium* YZS-M06 would markedly improve soil nutrient availability and enhance soil enzyme activities; (2) At the soil micro-ecological level, the successful colonization of functional bacteria would optimize soil bacterial community structure, facilitate the enrichment of beneficial microorganisms, and regulate nutrient transformation processes; (3) Under the synergistic ameliorative effects described above, the growth performance and stress resistance of the pioneer plant *Ipomoea pes-caprae* would be substantially enhanced. This study supports Sustainable Development Goal SDG 13 (Climate Action), 14 (Life Below Water), and 15 (Life on Land) by enhancing plant growth, improving soil health, restoring coastal ecosystems, and promoting sustainable biochar-microbe interventions (Fegade 2024a, b).

## Materials and methods

### Materials

The coastal sandy soil and *I. pes-caprae* employed in this study were collected from the Minjiang River Estuary Wetland in Fujian Province, China (E 119° 36′ 28″–E 119° 41′ 15″, N 26° 01′ 08 ″–N 26° 03′ 40″). Coastal sandy soil was sampled from the surface layer (0–20 cm) of the tidal flat within the wetland, air-dried, and passed through a 10-mesh sieve prior to use. The basic physicochemical properties of the soil were as follows: Soil basic properties: total nitrogen (TN) 0.11 ± 0.38 g/kg, ammonium nitrogen (NH_4_⁺-N) 0.14 ± 0.31 mg/kg, nitrate nitrogen (NO_3_⁻-N) 0.05 ± 0.03 mg/kg, available phosphorus 10.08 ± 0.02 mg/kg, available potassium 15.59 ± 4.39 mg/kg, available sulfur 18.52 ± 1.57 mg/kg, pH 5.75 ± 0.42, electrical conductivity (EC) 1.94 ± 0.52 mS/cm. The soil texture is loamy sand.

*F. velutipes* residues (obtained from Fujian Wanchen Biotechnology Group Co., Ltd.) were carbonized in a tube furnace (OTF-1200X) at 700 °C under a CO_2_ atmosphere for 2 h. After natural cooling, the samples were crushed, sieved through a 100-mesh sieve, washed with deionized water, and dried at 80 °C to constant weight for later use. The resulting biochar exhibited a pH of 10.38, ash content of 40.97%, volatile matter of 9.38%, nitrogen content of 1.82%, total carbon content of 52.41%, and C/N ratio of 28.91, specific surface area of 148.92 m²/g, the volume of the hole is 0.099 cm^3^/g and an average pore diameter of 2.28 nm.

*B. megaterium* YZS-M06 (CGMCC No. 20592) was used as the test strain to prepare the microbial inoculum. This strain was previously isolated by our research group and exhibits phosphate-solubilizing, potassium-solubilizing, and nitrogen-fixing capabilities. Inoculum preparation: A single colony of *B. megaterium* YZS-M06 was inoculated into nutrient broth medium and incubated at 28 °C with shaking at 150 r/min for 48 h, the bacterial suspension concentration was adjusted to 1 × 10⁸ CFU/mL using sterile deionized water. to obtain the *B. megaterium* YZS-M06 inoculant.

The prepared *B. megaterium* YZS-M06 inoculant was combined with *F. velutipes* residue-based biochar (sterilized at 121 °C for 20 min) at a bacteria-to-biochar ratio of 1:10 (v/w) in 250 mL Erlenmeyer flasks. The mixture was thoroughly agitated at 25 °C and 150 r/min for 24 h, followed by centrifugation at 25 °C and 1,500 r/min for 15 min. After removal of the supernatant, the precipitate was freeze-dried (D2F-6020) for 48 h and subsequently pulverized to obtain the *F. velutipes* residue biochar-based inoculant (Chantorn et al. 2022).

### Experimental design

The experiment comprised four treatments: control (CK, root irrigation with 6 mL of water only), biochar only (BC, application of 6 g *F. velutipes* residue-based biochar only, with a biochar-to-soil mixing ratio of 2% (w/w)), bacterial agent only (B, application of 6 mL *B. megaterium* YZS-M06 bacterial agent only, with a bacterial agent-to-soil mixing ratio of 2% (v/w)), and biochar-based bacterial agent (BCB, application of 6 g *F. velutipes* residue biochar-based bacterial agent, with a biochar-based bacterial agent-to-soil mixing ratio of 2% (w/w)).

Current-year *I. pes-caprae* plants were selected, and stem cuttings of uniform length between nodes were prepared, with each cutting retaining two nodes. To ensure morphological consistency, buds and leaves at the morphologically lower end were removed simultaneously. The cuttings were subsequently transplanted into cultivation pots (specifications: 9 × 8 × 7 cm, containing 300 g coastal sandy soil per pot) under the corresponding treatments, with five *I. pes-caprae* seedlings established per pot. Each treatment consisted of ten replicate pots to ensure adequate sample availability for destructive sampling while preserving experimental reliability. Biochar and biochar-based inoculants were evenly incorporated into the soil in powder form rather than applied as surface amendments. Deionized water was applied every 2 days with a small and frequent watering regime to ensure complete water infiltration without waterlogging, so as to avoid soil nutrient leaching caused by leakage.

Growth indices of *I. pes-caprae* were determined at 15 d and 30 d after the above treatments, respectively. Destructive sampling was conducted to collect newly formed leaves and rhizosphere soil samples. Treatment codes at 15 d: CK_1, BC_1, B_1, BCB_1; Treatment codes at 30 d: CK_2, BC_2, B_2, BCB_2. Each pot was regarded as an independent replicate. Newly formed leaves from 5 plants in each pot were combined into one leaf sample for that pot. Meanwhile, rhizosphere soil samples were collected from the same pot. Leaf and soil samples were homogenized separately and used as the final samples for each treatment, with three replicate pots per treatment used for analysis. Newly emerged leaves were harvested for the determination of leaf antioxidant enzyme activities, while the rhizosphere soil of *I. pes-caprae* was simultaneously collected according to the following procedures: after *I. pes-caprae* plants were carefully removed from the soil, loosely attached bulk soil was gently shaken off and discarded. Subsequently, the root system was placed into a sterile self-sealing bag and gently agitated to detach soil adhering to the root surface into the bag, which was defined as rhizosphere soil. For rhizosphere soil samples obtained from each treatment group, one portion was stored at 4 °C for soil enzyme activity analysis; a second portion was rapidly frozen in liquid nitrogen and preserved at − 80 °C for subsequent DNA extraction; and the remaining portion was air-dried at room temperature for the determination of soil chemical properties.

### Determination of soil chemical properties and enzyme activities

Soil chemical properties were determined (three replicates per sample) (Gu et al. 2019; Liu et al. 2024). APwas extracted using the HClO_4_–H_2_SO_4_ digestion method, and the contents of AP and total phosphorus (TP) were quantified using a SAN + + continuous flow analyzer (Skalar, Netherlands). AKwas extracted with 1.0 mol L^−1^ CH_3_COONH_4_ solution, and its concentration was measured using a PinAAcle 900 H flame atomic absorption spectrometer (PERKINELMER, USA). Prior to nitrogen analysis, soil samples were pretreated with 0.5 mol L^−1^ HCl to eliminate inorganic carbonates, after which NH_4_⁺–N and NO_3_⁻–N contents were determined using the indophenol blue method and the nitrosalicylic acid method, respectively. AS content was determined by barium sulfate turbidimetry. Total nitrogen (TN) and total potassium (TK) were extracted using the HNO_3_–HF–HCl digestion method and subsequently measured by an inductively coupled plasma optical emission spectrometer (AVIO 200).

Soil urease (S-UE) activity was determined using indophenol blue colorimetry (Guo et al. 2012), soil acid phosphatase (S-ACP) activity was quantified by phenol colorimetry (Hou et al. 2020), and soil leucine aminopeptidase (S-LAP) activity was measured using p-nitroaniline colorimetry (Saiya-Cork et al. 2002).

### Soil DNA extraction and microbial sequencing analysis

An accurately weighed 0.5 g soil sample was subjected to DNA extraction using the E.Z.N.A.® Soil DNA Kit (Omega Bio-tek, USA); after the extracted DNA met the quality criteria (total amount ≥ 15 µg, concentration ≥ 50 ng·µL^−1^), the V3–V4 hypervariable regions of the bacterial 16 S rRNA gene were amplified with the universal bacterial primers 338 F (5′-ACTCCTACGGGAGGCAGCAG-3′) and 806R (5′-GGACTACHVGGGTWTCTAAT-3′) in a 50 µL PCR reaction system consisting of 5 µL of 10× PCR buffer, 5 µL of 2 mmol·L^−1^ dNTPs, 1 µL of DNA polymerase, 1.5 µL each of forward and reverse primers, 50 ng of DNA template, and the remaining volume supplemented with double-distilled water, with the PCR cycling program set as follows: initial denaturation at 94 °C for 10 min, followed by 35 cycles of denaturation at 94 °C for 30 s, annealing at 56 °C for 30 s and extension at 72 °C for 30 s, and a final extension step at 72 °C for 10 min; each sample was amplified in triplicate with a no-template control (NTC) included in the system, the quality of PCR amplicons was then assessed using the QuantiFluor™-ST Blue Fluorescence Quantification System (Promega, USA), and qualified amplicons were sequenced on the Illumina MiSeq platform by Majorbio Bio-pharm Technology Co., Ltd. (Shanghai, China).

### Determination of growth indicators and leaf antioxidant enzyme activities of *Ipomoea pes-caprae*

Growth indicators of *I. pes-caprae* were determined (with three replicates per sample). The entire plant was thoroughly washed, surface moisture was carefully blotted dry, and fresh weight was recorded. Subsequently, the plant material was placed in a kraft paper bag and oven-dried at 75 °C until a constant weight was achieved, after which the dry weight was measured. The numbers of shoots and roots of *I. pes-caprae* were quantified by direct counting, whereas shoot length, plant height, and root length were measured using a ruler.

Determination of enzyme activities in fresh leaves of *I. pes-caprae* (3 replicates). Leaf catalase (CAT) activity was quantified using the ammonium molybdate method (Lian et al. 2023), leaf peroxidase (POD) activity was determined using the guaiacol method (Hu et al. 2023), and leaf superoxide dismutase (SOD) activity was assessed based on the xanthine and xanthine oxidase reaction system that generates superoxide anion (O_2_^−^), which reacts with water-soluble tetrazolium salt-1 to form water-soluble yellow formazan (Qiu et al. 2023). Additionally, leaf malondialdehyde (MDA) content was determined via the condensation reaction between MDA and thiobarbituric acid under acidic and high-temperature conditions (Xu et al. 2023), and leaf soluble sugar content was measured using the anthrone colorimetric method (Du et al. 2023).

### Statistical analysis

Excel 2019 and SPSS 23.0 were used for data preprocessing and variance analysis of plant growth parameters, antioxidant enzyme activities, soil chemical properties, and soil enzyme activity indicators, followed by post-hoc comparisons (One-way ANOVA + Fisher LSD, *p* < 0.05).

Flash v1.2.11 software was applied for paired-end sequence assembly to obtain complete gene sequences (Magoč and Salzberg 2011). RDP Classifier v2.13 software was employed for operational taxonomic unit (OTU) clustering and annotation to acquire taxonomic information of the sequences (Wang et al. 2007). Usearch v11 software was utilized to enumerate OTUs and calculate microbial relative abundance (Edgar 2010), whereas Qiime v1.9.1 software was used to generate microbial absolute abundance tables. Principal component analysis based on OTU counts was performed using the vegan package (v2.7-2) in R v4.5.1 (analyzed via the FactoMineR method), and graphical visualization was conducted using the ggplot2 v3.3.3 package (v3.3.3) in R v4.5.1 (Warton et al. 2012). Spearman correlation analysis was conducted using the stats package (v4.5.1) in R v4.5.1, with visualization carried out through the pheatmap package (v1.0.13) in R v4.5.1. Functional prediction of bacterial communities was performed using FAPROTAX v1.2.1. Topological characteristic indices were calculated using the ggClusterNet package (v2.0) in R v4.5.1 (Ma et al. 2016), and co-occurrence network visualization was conducted using the ggNetView package (v0.1.0). Partial least squares path modeling (PLS-PM) structural equation model fitting was performed using the plspm package (v0.4.9) in R v4.5.1.

## Results

### Effects of FV-biochar loaded with *Bacillus megaterium* on soil chemical properties and enzyme activities

#### Soil chemical properties

**Fig. 1:**
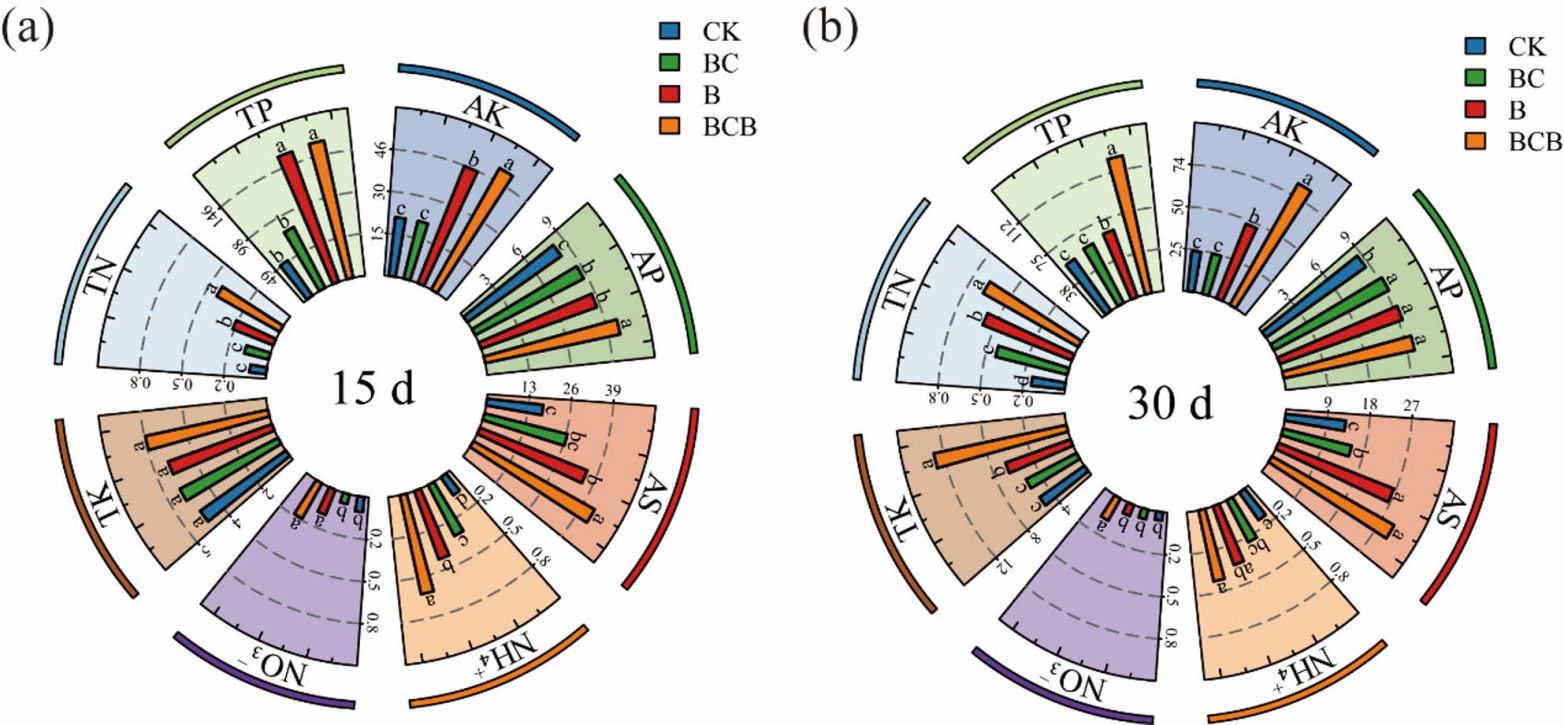
Effects of FV-biochar loaded with *Bacillus megaterium* on soil chemical indicator contents. **a** Represents the soil chemical indicators of the CK, BC, B, and BCB treatments at 15 days; **b** Represents the soil chemical indicators of the CK, BC, B, and BCB treatments at 30 days. Different lowercase letters in the figure indicate significant differences among groups (*p <* 0.05)

Soil chemical indicators were significantly enhanced under BCB treatment, with substantial increases observed relative to CK treatment at both 15 and 30 days. However, the magnitude of increases in TP and ammonium nitrogen decreased over time, whereas the enhancements in AK and nitrate nitrogen were further intensified (Fig. 1, Table S1). For nitrogen, the total nitrogen content under BCB treatment was significantly higher than that under CK (0.10, 0.20 g/kg, *p <* 0.05) at 15 d (0.43 g/kg) and 30 d (0.65 g/kg). Ammonium nitrogen peaked at 15 d (0.60 mg/kg), which was significantly higher than that under other treatments. Nitrate nitrogen was significantly higher than that under CK (0.09, 0.05 mg/kg, *p <* 0.05) at 15 d (0.24 mg/kg) and 30 d (0.15 mg/kg). For phosphorus, total phosphorus under BCB treatment was significantly higher than that under CK (49.93, 56.60 mg/kg, *p <* 0.05) at 15 d (162.33 mg/kg) and 30 d (124.91 mg/kg). Available phosphorus was significantly higher than that under CK (8.19, 8.69 mg/kg, *p <* 0.05) at 15 d (9.83 mg/kg) and 30 d (9.52 mg/kg). For potassium, available potassium under BCB treatment was significantly higher than that under other treatments at 15 d (50.42 mg/kg). At 30 d, both total potassium (12.89 g/kg) and available potassium (81.75 mg/kg) reached the highest values, which were significantly higher than those under B (6.58 g/kg, 47.06 mg/kg) and CK (5.20 g/kg, 23.77 mg/kg, *p <* 0.05). For sulfur, available sulfur under BCB treatment was significantly higher than that under CK (17.46, 12.94 mg/kg, *p <* 0.05) at 15 d (42.94 mg/kg) and 30 d (29.96 mg/kg).

Other treatments also resulted in improvements in soil nutrient status to varying extents. Among these, B treatment generally exhibited superior performance relative to BC treatment for most indicators, particularly demonstrating greater increases in TK, TN, and AK at 30 days. BC treatment showed moderate enhancement of certain indicators, such as TP and ammonium nitrogen at 15 days. However, the overall magnitude and persistence of these effects were comparatively limited.

#### Soil enzyme activities

**Fig. 2:**
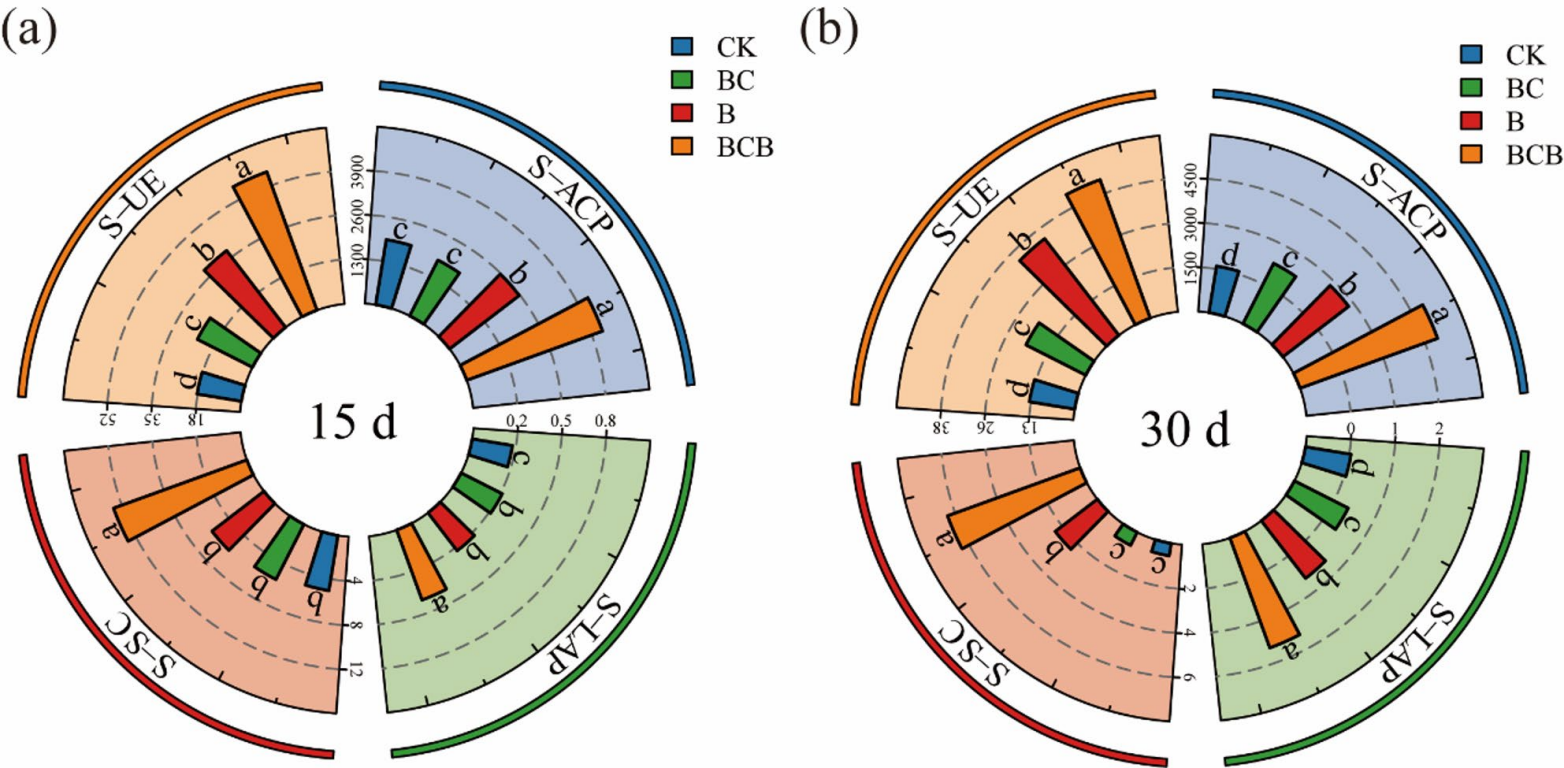
Effects of FV-biochar loaded with *Bacillus megaterium* on soil enzyme activities. **a** Represents the soil enzyme activity indicators of the CK, BC, B, and BCB treatments at 15 days; **b** Represents the soil enzyme activity indicators of the CK, BC, B, and BCB treatments at 30 days. Different lowercase letters in the figure indicate significant differences among groups (*p* < 0.05)

Soil enzyme activities, including S-UE, S-ACP, and S-LAP, were significantly enhanced under BCB treatment (Fig. 2, Table S2). At 15 and 30 days of BCB treatment, S-UE activities reached 58.13 U/g and 42.39 U/g, respectively, corresponding to increases of 228% and 221% relative to CK. S-LAP activities were recorded at 0.42 U/g and 1.32 U/g, representing increases of 83% and 154%, respectively. S-ACP activities increased to 4235.48 U/g and 4949.05 U/g, with increases of 119% and 210%, respectively, demonstrating that BCB treatment consistently and markedly elevated all three soil enzyme activities.

Across the remaining treatments, the B treatment exhibited significantly greater enhancement of all three enzyme activities than the BC treatment, with particularly pronounced improvements observed for S-UE and S-ACP. Although BC treatment resulted in measurable increases in enzyme activities, the overall magnitude of enhancement was lower than that achieved by B treatment. Therefore, the overall effectiveness of the treatments on soil enzyme activities followed the order: BCB > B > BC > CK.

### Effects of FV-biochar loaded with *Bacillus megaterium* on soil micro-ecology

#### Quality of bacterial community sequencing

Sequencing results showed that a total of 1,792,238 raw reads were obtained for the V3–V4 region, which were filtered to 1,571,199 reads after sequence quality control and optimization. After removing unclassified taxa, a total of 33 phyla, 83 classes, 167 orders, 205 families, 359 genera, and 829 species were detected. As shown in the rarefaction curve (Fig. S1), the number of detectable species gradually plateaued with increasing sequence numbers per sample, indicating that the detected species were sufficient to represent the bacterial community composition in the samples.

#### Changes in soil bacterial abundance and activity

**Fig. 3:**
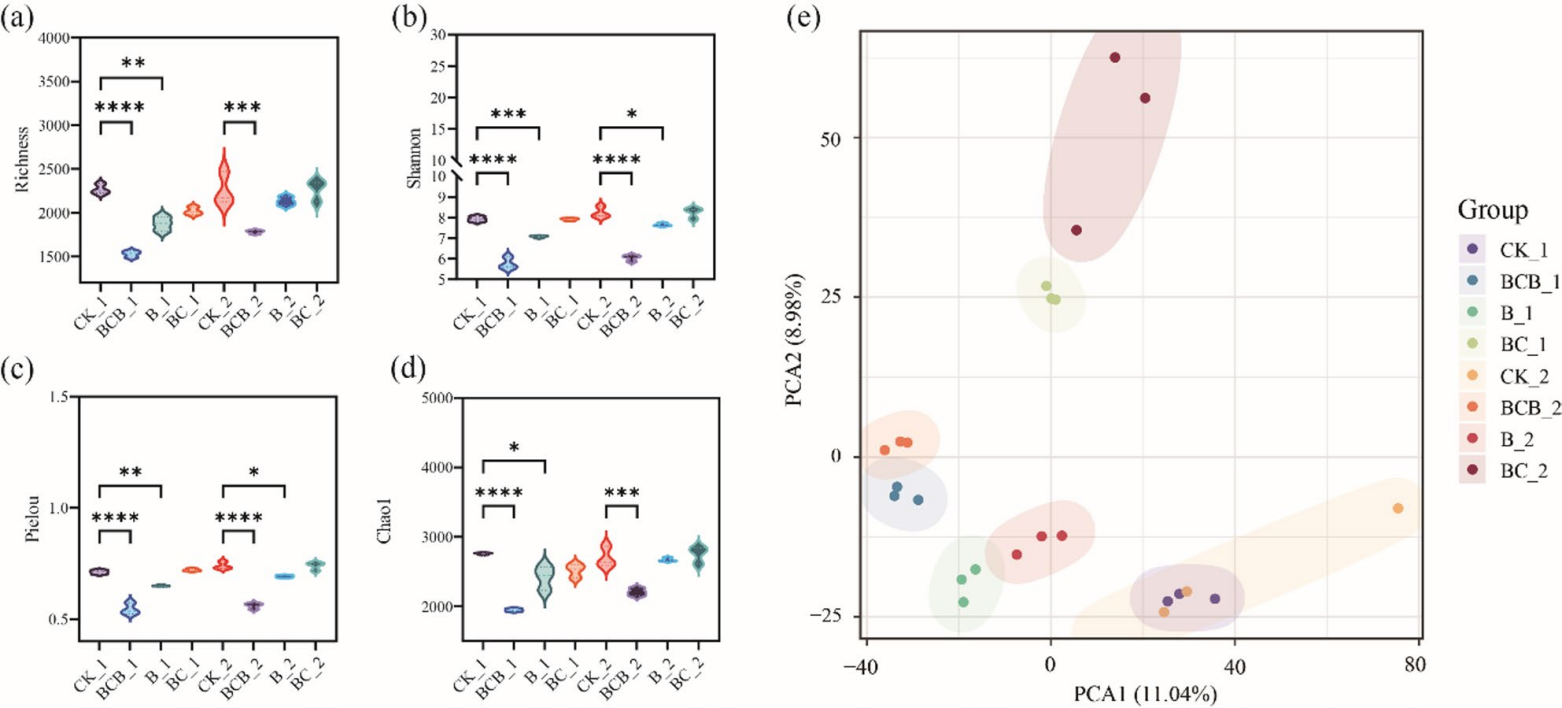
Alpha diversity and beta diversity. **a** Shows the richness index of bacterial communities under CK, BC, B, and BCB treatments; **b** Shows the Shannon index of bacterial communities under CK, BC, B, and BCB treatments; **c** Shows the Pielou index of bacterial communities under CK, BC, B, and BCB treatments; **d** Shows the Chao1 index of bacterial communities under CK, BC, B, and BCB treatments; **e** Shows the principal component analysis of bacterial communities under CK, BC, B, and BCB treatments. “*” indicates significant differences between groups, *p* < 0.05; “**” indicates significant differences between groups, *p* < 0.01; “***” indicates significant differences between groups, *p* < 0.001; “****” indicates significant differences between groups, *p* < 0.0001;

Alpha diversity analysis (Table S3) indicated that Richness, Shannon, Pielou, and Chao1 indices were significantly reduced under the BCB_1 treatment compared with the CK_1 treatment (*p* < 0.05), and this pattern was maintained at 30 days. A comparable trend was observed for the B treatment. However, by day 30, the effects on Richness and Chao1 indices were no longer statistically significant (*p* > 0.05). Throughout the experimental period, no significant variations in alpha diversity indices were detected under the CK treatment, In the principal component analysis (PCA), in which the confidence ellipses for days 15 and 30 under CK nearly overlapped (Fig. 3), indicating the absence of significant differences in rhizosphere bacterial community structure within the CK treatment during the experimental cycle. In contrast, significant shifts in bacterial community composition were detected across all other treatment groups.

#### Changes in soil microbial community structure

**Fig. 4:**
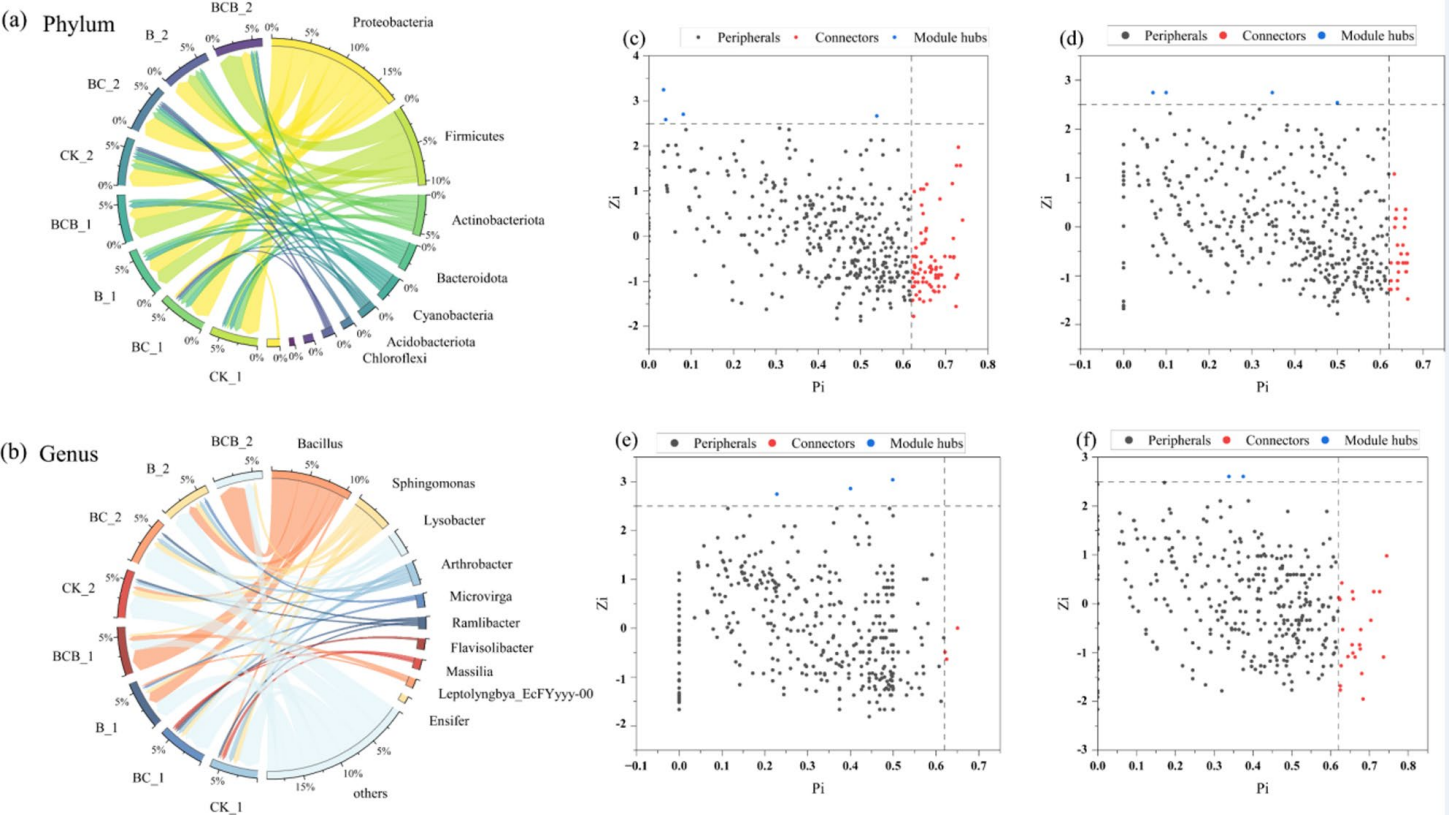
Top 10 relative abundance at phylum and genus levels and ZiPi keystone species analysis. **a** Represents the abundance of dominant bacterial phyla in the bacterial community under CK, BC, B, and BCB treatments; **b** Represents the abundance of dominant bacterial genera in the bacterial community under CK, BC, B, and BCB treatments; **c** Shows the screening situation of core bacterial genera under CK treatment; **d** Shows the screening situation of core bacterial genera under BC treatment; **e** Shows the screening situation of core bacterial genera under B treatment; **f** Shows the screening situation of core bacterial genera under BCB treatment

Distinct effects of the different treatments on soil microbial community structure were observed, with BCB treatment preferentially enriching Firmicutes and Bacillus. From day 15 to day 30 across all treatments, at the phylum level (Fig. 4a, Table S4), Proteobacteria maintained a relatively high abundance under BC treatment (45.81%→45.00%), whereas the relative abundance of Firmicutes under BCB treatment increased from 43.34% to 49.77%. In contrast, Actinobacteriota exhibited a decline under CK treatment (15.66%→13.13%), and Cyanobacteria decreased under BCB treatment (11.86%→6.58%), no significant differences were observed in the above phylum changes (*p <* 0.05). At the genus level (Fig. 4b, Table S5), *Bacillus* was enriched under BCB treatment significantly (46.58%→57.85%, *p* < 0.05), *Sphingomonas* exhibited a slight increase under CK treatment (12.86%→14.00%), *Lysobacter* declined under BC treatment significantly (11.75%→8.43%, *p* < 0.05), and *Arthrobacter* increased under BC treatment (8.10%→9.10%).

Microbial co-occurrence network analysis based on Zi–Pi parameters indicated that soil microbial community structure was differentially influenced by the various treatments (Fig. 4). The network associated with CK treatment was relatively simple and predominantly composd of peripheral nodes (98.3%), with only four module hubs and ten connectors identified. By comparison, B treatment enhanced inter-module connectivity, as evidenced by a 60% increase in the number of connectors to 16, despite a reduction in module hubs to three, suggesting that its primary influence was associated with facilitating inter-module interactions rather than reinforcing intra-module organization. In contrast, BC treatment promoted the formation of core nodes within modules, with the number of module hubs increasing to seven while maintaining fourteen connectors, thereby substantially enhancing network complexity and stability. Notably, BCB treatment exhibited synergistic effects rather than simple additive effects, reshaping the functional roles of keystone species and generating new keystone taxa, such as *Caulobacte*r (serving as a module hub under BCB treatment while remaining a peripheral node in other treatments), ultimately resulting in a network structure that was more optimized with respect to both module regulation and inter-module coordination.

#### Contribution analysis of soil amelioration

**Fig. 5:**
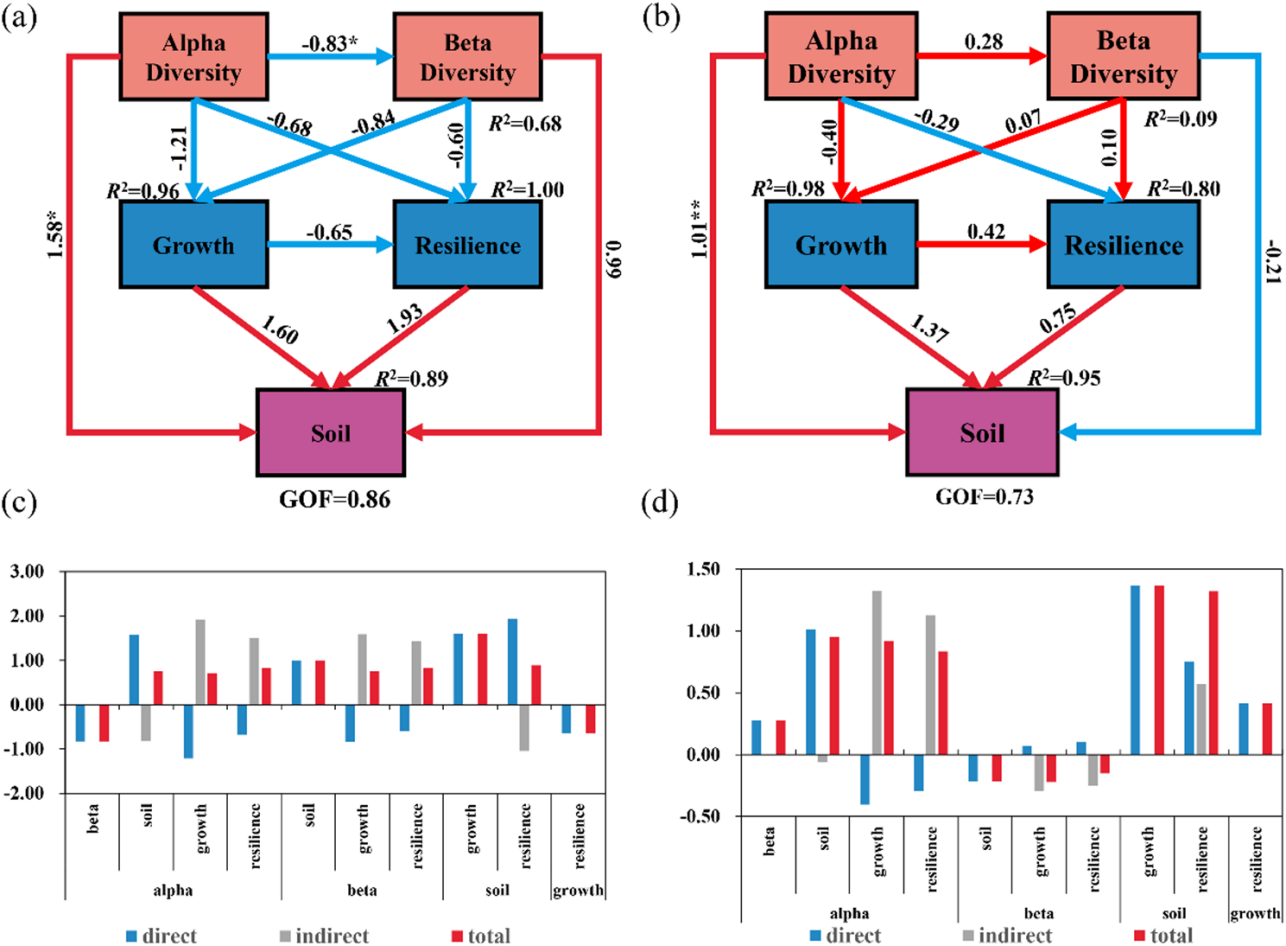
PLS-PM structural equation model. Path analysis model illustrating the effects of bacterial community alpha diversity and beta diversity on plant growth and resilience, and ultimately on soil properties. Arrows indicate the direct effects of independent variables on dependent variables, with values on the paths representing standardized regression coefficients that indicate the direction and strength of the effects. Model fit is represented by the coefficient of determination (R²) and the goodness-of-fit index (GQF). Asterisks denote the significance levels of the paths: “*” indicates a significant effect (*p* < 0.05); “**” indicates a highly significant effect (*p* < 0.01). Panels: **a** CK Treatment; **b** BCB treatment; **c** Direct, indirect, and total effect coefficients among variables under CK treatment; **d** Direct, indirect, and total effect coefficients among variables under BCB treatment

To comprehensively examine the interactions among soil, microorganisms, and plants, PLS-PM was employed in this study. Five latent variables, including alpha diversity, beta diversity, growth, stress resistance, and soil factors, were established, and previously measured soil, plant, and bacterial indicators were screened according to manifest variable loadings (≥ 0.7) to construct the structural model. The results demonstrated that, in comparison with the CK treatment (Fig. 5a), the BCB treatment (Fig. 5b) markedly altered the relationships among multiple pathways. Specifically, the effects of alpha diversity—beta diversity, alpha diversity—growth, beta diversity—growth, beta diversity—stress resistance, and growth—stress resistance shifted from negative to positive. In contrast, the relationship between beta diversity and soil factors changed from a positive to a negative influence. As illustrated in Fig. 5c and d, the direction of the influence of alpha diversity on beta diversity differed entirely between the two treatments. Under the BCB treatment, a positive effect was observed, with a path coefficient of 0.279, whereas a significant negative effect was detected under the CK treatment (− 0.830). With respect to the soil factor pathway, alpha diversity under the BCB treatment exerted a significant positive direct effect on soil factors (1.014, *p* < 0.01). Although a stronger direct effect was observed under the CK treatment (1.580), its total effect was lower than that of the BCB treatment due to offsetting by a negative indirect effect mediated through beta diversity. Additionally, soil factors under the BCB treatment promoted plant growth (1.366), whereas this pathway exhibited a stronger effect under the CK treatment (1.600). Simultaneously, growth exerted a positive influence on stress resistance under the BCB treatment (0.417), while a negative influence was observed under the CK treatment (− 0.652).

**Fig. 6:**
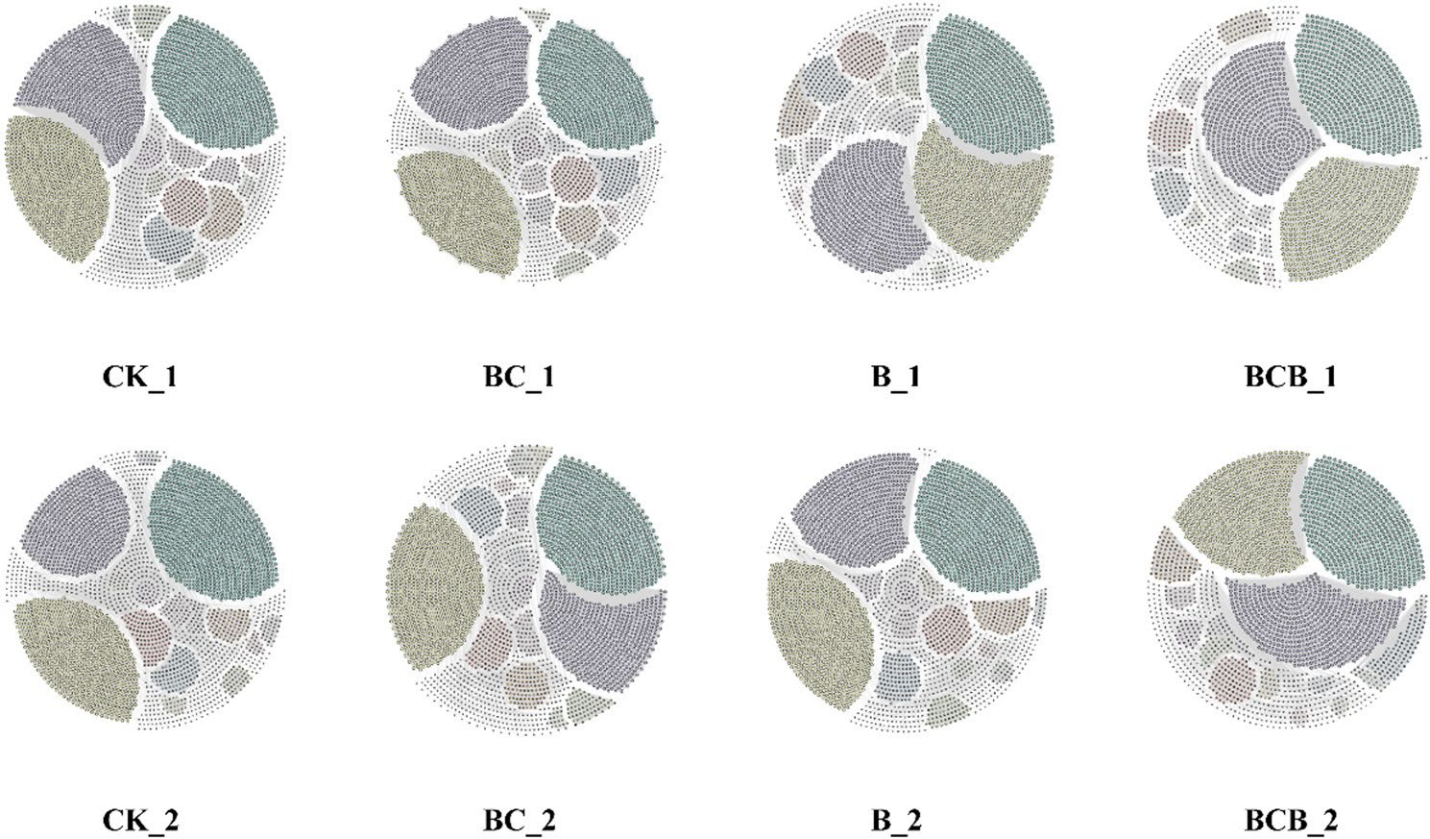
Network diagram. Bacterial co-occurrence networks under CK, BC, B, and BCB treatments at 15 days (denoted by the suffix “_1”) and 30 days (denoted by the suffix “_2”)

Concurrently, based on analyses of network topological characteristics (Table S6) and network structure (Fig. 6), the highest degree of centralization (0.08) was observed under the BCB_1 treatment. With increasing cultivation time, the degree of centralization increased to varying extents across all treatments except CK. With respect to modularity, relatively high values were recorded for the B_1 and BC_1 treatments, at 9.47 and 9.65, respectively. Notably, the modularity of the B_2 treatment further increased to 9.64. In contrast, network modularity under the CK treatment exhibited a pronounced decline with extended cultivation time, decreasing from an initial value of 8.62 to 7.91.

**Fig. 7:**
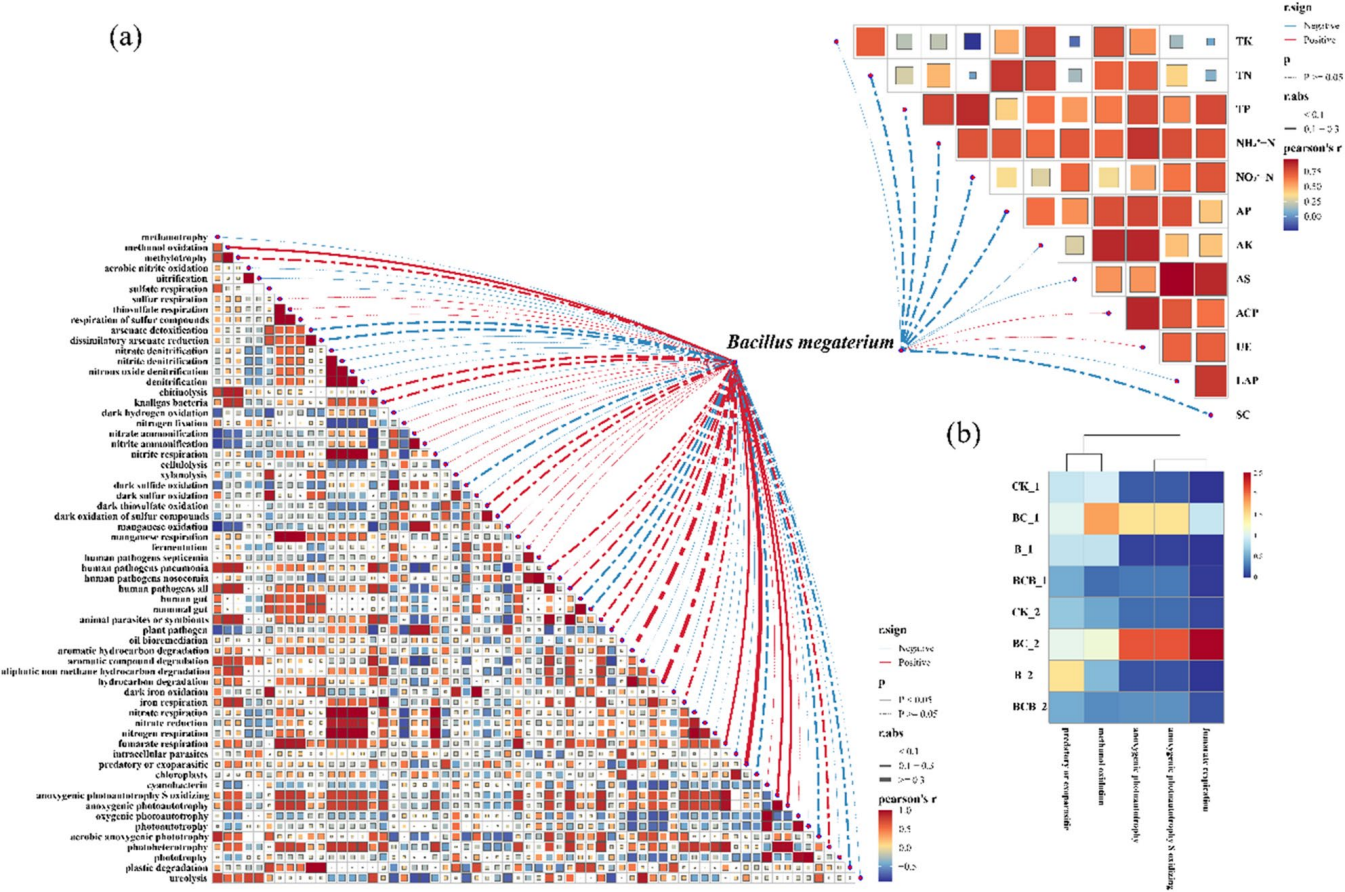
Correlation network among *Bacillus megaterium*, soil properties, functional predictions, and enzyme activities. a Co-occurrence network illustrating the interactions among *B. megaterium* YZS-M06, the relative abundance of functional bacterial communities, soil chemical properties, and soil enzyme activities. **b** Correlation analysis showing the expression abundance of functions significantly associated (*p* < 0.05) with *B. megaterium* M06 under CK, BC, B, and BCB treatments at 15 days (denoted by the suffix “_1”) and 30 days (denoted by the suffix “_2”)

To elucidate the effects of *B. megaterium* YZS-M06 on soil chemical properties, plant growth and stress resistance, and functional bacterial communities, Mantel tests were applied to evaluate correlations between this strain and the aforementioned indicators (Fig. 7). Functional prediction analysis results, based on the Kruskal–Wallis rank-sum test and Tukey–Kramer multiple comparisons (*p* < 0.05) with false discovery rate correction, indicated that *B. megaterium* exhibited significant positive correlations (*p* < 0.05) with multiple microbial functions, including methanol oxidation, fumarate respiration, predatory or exoparasitic activity, and anoxygenic photoautotrophy. Further analysis demonstrated that, among the different treatments, the BC treatment displayed the strongest associations with these functions, and that these correlations progressively intensified over time (Fig. 7).

### Effects of FV-biochar loaded with *Bacillus megaterium* on the growth and physiological characteristics of *Ipomoea pes-caprae*

#### Growth performance of Ipomoea pes-caprae

**Fig. 8:**
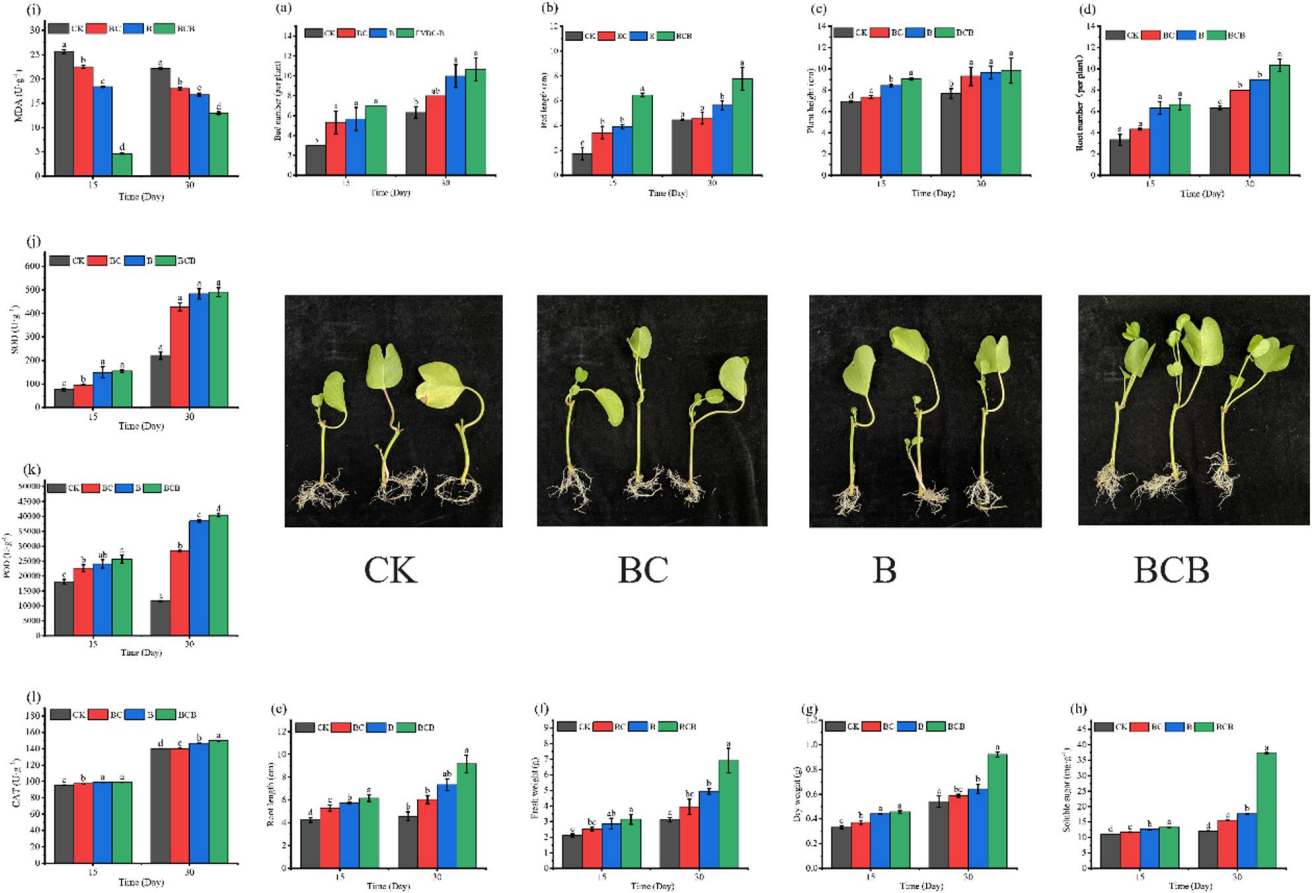
Effects of FV-biochar loaded with *Bacillus megaterium* on **a** bud number, **b** bud length, **c** plant height, **d** root number, **e** root length, **f** fresh weight, **g** dry weight, **h** soluble sugar content, **i** MDA content, **j** SOD, **k** POD, and **l** CAT activities of *Ipomoea pes-caprae*

As illustrated in Fig. 8 and Table S7, at both 15 and 30 days, all treatments resulted in higher values than the CK treatment with respect to bud number, bud length, plant height, root number, and root length of *I. pes-caprae*, with the overall performance ranking as BCB > B > BC > CK. At 15 days of treatment, the BCB treatment produced the highest values across all growth indicators, with bud number, bud length, plant height, root number, and root length significantly increased by 133%, 273%, 31%, 100%, and 45%, respectively, relative to the CK treatment (*p* < 0.05). At 30 days, the BCB treatment remained the most effective, with significant increases of 68%, 74%, 28%, 63%, and 101% in bud number, bud length, plant height, root number, and root length, respectively, compared with the CK treatment (*p* < 0.05).

At 15 days, the B treatment significantly increased bud number, bud length, and plant height of *I. pes-caprae* by 88.9%, 125%, and 21.6%, respectively, relative to the CK treatment (*p* < 0.05). Similarly, the BC treatment resulted in significant increases in bud number and bud length of 77.8% and 98.0%, respectively, compared with the CK treatment (*p* < 0.05). By 30 days, the B treatment led to significant increases in bud number, root number, and root length relative to the CK treatment, with increments of 57.9%, 42.1%, and 60.6%, respectively (*p* < 0.05). Similarly, the BC treatment significantly increased bud number, root number, and root length by 26.3%, 26.3%, and 32.1%, respectively, compared with the CK treatment (*p* < 0.05) (Table S7) .

#### Antioxidant enzyme activities and stress resistance of Ipomoea pes-caprae leaves

The accumulation of osmotic adjustment substances and the antioxidant enzyme system in *I. pes-caprae* were significantly enhanced under BCB treatment, thereby improving stress resistance. The results indicated that BC, B, and BCB treatments all significantly increased soluble sugar content as well as SOD, POD, and CAT activities in *I. pes-caprae*, whereas MDA content was significantly reduced (*p* < 0.05) (Fig. 8, Table S8). At 15 days, soluble sugar content and SOD, POD, and CAT activities under BCB treatment were increased by 20%, 105%, 247%, and 4%, respectively, relative to CK treatment, while MDA content was significantly decreased by 82% (*p* < 0.05). By 30 days, these physiological indicators under BCB treatment were further strengthened, with soluble sugar content and SOD and POD activities significantly increased by 208%, 123%, and 42%, respectively, compared with CK treatment, whereas MDA content was significantly reduced by 42% (*p* < 0.05). Collectively, BCB treatment exhibited superior performance in promoting osmotic adjustment substance accumulation and enhancing the antioxidant enzyme system of *I. pes-caprae* relative to B and BC treatments.

## Discussion

This study adopts a green approach by converting agricultural waste into biochar and combining it with beneficial *B. megaterium*, applying the the principles of waste prevention, use of renewable feedstocks, and environmentally friendly biological catalysis as proposed and clarified in the recent study (Fegade 2024c).

### FV-biochar loaded with *Bacillus megaterium* improves soil fertility and ecological functions

Studies have shown that BCB treatment can effectively improve soil chemical properties. Specifically, soil total potassium, total nitrogen, total phosphorus, ammonium nitrogen, nitrate nitrogen, available phosphorus, available potassium, and available sulfur were all significantly higher than those in the CK treatment, BC treatment, and B treatment. The results of this study are also consistent with those reported by Teng et al. (2024) regarding the improvement of soil nutrients. Biochar loaded with microorganisms is superior to biochar alone and functional bacteria alone in improving soil chemical properties. Hou et al. (2025) found in coastal saline-alkali soil that biochar combined with *Azotobacter chroococcum* further increased soil available nitrogen content by 12.8% compared with the single inoculant treatment. This enhancement may be attributed to the capacity of microorganisms to metabolically produce nitrogenase, phosphatase, nuclease, or organic acids, thereby facilitating nitrogen fixation and phosphorus solubilization (Olaniyan et al. 2022). Additionally, biochar increases soil cation exchange capacity and strengthens interactions with microorganisms, promoting nutrient retention through effective adsorption and reducing leaching losses of nutrients such as N, P, and K, Lu et al. found in a soil column leaching experiment that biochar application reduced soil phosphorus leaching loss by 20.5%–47.1% (Lu et al. 2021). Therefore, biochar loaded with microorganisms is able to exert synergistic effects between the two components, facilitating nutrient uptake and utilization by plants and ultimately contributing to improved soil fertility (Md. Nasir Hossain et al. 2020).

Soil enzyme activity serves as an indicator of microbial activity and nutrient transformation processes (Meena et al. 2024). In this study, soil enzyme activity was effectively enhanced by *F. velutipes* residue-based biochar loaded with *B. megaterium*. This enhancement may be attributed to the ability of biochar to stimulate microbial metabolism and elevate microbial activity by supplying intrinsic carbon sources and nutrients to the soil matrix (Luo et al. 2020). Additionally, microorganisms such as plant growth-promoting rhizobacteria contribute to increased soil enzyme activity by providing substrates for enzymatic reactions and facilitating organic matter decomposition (Azeem et al. 2021). Previous studies have indicated that biochar loaded with microorganisms can elevate soil nutrient levels and microbial diversity, thereby promoting enzyme activity (Jabborova et al. 2024). On the one hand, biochar, characterized by a porous structure, high specific surface area, and abundant surface functional groups, provides a favorable habitat and protective microenvironment for microorganisms, thereby supporting microbial colonization, persistence, and metabolic activity. On the other hand, microorganisms directly participate in the transformation and release of soil nutrients, including nitrogen, phosphorus, and potassium, through the secretion of metabolic products such as nitrogenase, phosphatase, nuclease, and organic acids, while biochar can adsorb these enzymes and metabolites, thereby extending their functional longevity in soil. Meanwhile, as a typical plant growth-promoting rhizobacterium, *B. megaterium* can dissolve insoluble phosphate in soil by secreting organic acids (e.g., gluconic acid), convert unavailable phosphorus into available forms absorbable by plants, and produce extracellular enzymes such as phosphatase and nuclease, which directly participate in the mineralization of organic phosphorus, thereby improving the bioavailability of soil nutrients (Sojka et al. 2025). Compared with previous studies using *B. megaterium* alone (Yang et al. 2025), the immobilization technology adopted in this study further enhanced the colonization ability and enzymatic stability of microorganisms, thus exhibiting a more significant effect in improving soil fertility.

### FV-biochar loaded with *Bacillus megaterium* can optimize soil micro-ecology

#### FV-biochar loaded with *Bacillus megaterium* YZS-M06 synergistically drives optimization of soil bacterial communities

By integrating the findings of this study with existing literature, the significant inhibitory effect of *F. velutipes* residue-based biochar loaded with *B. megaterium* on the alpha diversity of bacterial communities in the *I. pes-caprae* rhizosphere was determined to be a reproducible phenomenon rather than an isolated case. Consistently, Wang et al. (2025) in an investigation of the effects of different carbon materials on rice rhizosphere bacterial communities, reported that biochar-type materials generally reduce community richness and evenness. This pattern is in agreement with the present results, in which BC treatment failed to induce significant changes, whereas BCB treatment markedly altered community structure, indicating that the synergistic interaction between biochar and *B. megaterium* YZS-M06 represents a primary driver of community optimization. Mechanistically, this response may be attributed to the rapid colonization of functional microorganisms introduced by *F. velutipes* residue-based biochar loaded with *B. megaterium* within the rhizosphere. Such colonization suppresses the proliferation of certain indigenous microbial populations through intensified competition for nutrients and ecological niches, thereby resulting in reduced community evenness and richness (Wu et al. 2020). In contrast, the relatively narrow gradient of microbial community shifts induced by BC treatment in the surrounding soil, with effects largely confined to the regulation of environmental parameters such as pH and inorganic nitrogen, was insufficient to substantially modify overall community structure. This observation indicates that the regulatory role of biochar is primarily manifested through modulation of soil physicochemical properties rather than through direct restructuring of microbial composition. Furthermore, the attenuated influence of the B_2 treatment on Richness and Chao1 indices suggests that the ecological effects of a single microbial strain may be progressively buffered by environmental adaptation over time (Schuster et al. 2001).

#### Specific responses of microbial community composition at phylum and genus levels and keystone species

Distinct treatments exerted differentiated shaping effects on soil microbial community structure. At the phylum level, the sustained high abundance of *Proteobacteria* under BC treatment and the significant enrichment of Firmicutes under BCB treatment suggested that distinct dominant phyla were selectively favored through treatment-induced alterations in soil microenvironmental conditions or nutrient availability. Among them, the increase in Firmicutes (with its dominant genus Bacillus also significantly enriched) in the BCB treatment was mainly derived from the introduction of exogenous microbial agents. Meanwhile, the microenvironment created by biochar may also favor the colonization and growth of this bacterial community to a certain extent, which jointly contributed to the significant increase in its abundance. More importantly, co-occurrence network analysis based on Zi–Pi parameters demonstrated pronounced treatment-specific effects from the perspective of ecological network topology. Under CK treatment, a relatively simple network structure dominated by peripheral nodes was observed, reflecting a lower level of connectivity within microbial communities under minimally disturbed conditions. In contrast, B treatment resulted in a substantial increase in the number of connectors, consistent with previous findings indicating that microbial inoculants can function as “bridges” facilitating interspecies communication among distinct functional modules (Lei et al. 2020). This pattern may arise from the establishment of novel interactions between inoculated strains and indigenous microbial populations, thereby enhancing inter-module connectivity. Conversely, BC treatment led to a significant increase in the number of module hubs, strongly supporting the concept that biochar can promote the formation of critical core species within modules by offering diversified microhabitats through its porous structure (Lei et al. 2019). Furthermore, BCB treatment reshaped the composition of keystone species, resulting in the emergence of *Caulobacter* as a new module hub. It was postulated that biochar, acting as a physical carrier, enhanced the colonization and persistence of *B. megaterium* YZS-M06, thereby generating a more favorable microenvironment for its proliferation. Simultaneously, biochar-mediated regulation of soil chemical properties and the biological functions contributed by *B. megaterium* YZS-M06 interacted synergistically, collectively constructing a network architecture characterized by improved balance in module regulation and inter-module coordination, and exhibiting greater theoretical stability.

#### FV-biochar loaded with *Bacillus megaterium* reshapes key ecological pathways and drives functional optimization of rhizosphere microbial communities

Relative to the CK treatment, BCB treatment resulted in the reversal of several key pathway relationships from negative to positive, including alpha diversity—beta diversity, alpha diversity—growth, and beta diversity—stress resistance. This transformation indicated that niche optimization was achieved under BCB treatment through the promotion of community functional differentiation, which is consistent with previous reports on the synergistic enhancement of plant growth by biochar-loaded functional bacteria (Wang et al. 2025). Network analysis further demonstrated that *F. velutipes* residue-based biochar loaded with *B. megaterium* YZS-M06 exhibited the highest degree of centralization (0.082) at 15 days of treatment, with centralization showing an overall increasing trend over time. Meanwhile, modularity characteristics displayed divergent evolutionary patterns among treatments, thereby driving functional reorganization of microbial communities through the selective enrichment of key taxa and optimization of microbial interaction network architecture (Lin et al. 2016). A distinctive outcome of this study was that BCB treatment not only modified the magnitude of path coefficients but also reversed the direction of critical ecological pathways. This form of “relationship reshaping” exhibited clear advantages over treatments involving biochar or microbial inoculants applied individually.

### Synergistic mechanisms of growth promotion and stress resistance between FV-biochar loaded with *Bacillus megaterium*

#### FV-biochar loaded with *Bacillus megaterium* significantly enhanced growth promotion effects

BCB treatment performed the best in terms of the growth indices of *I. pes-caprae*, indicating a clear synergistic growth-promoting effect between biochar and *B. megaterium*. The porous structure of biochar provides an ideal habitat for *B. megaterium* (Zhang et al. 2025), significantly prolonging the active duration of functional bacteria in soil. Studies have shown that loading bacilli onto biochar can effectively avoid the adverse effects of water-soluble inhibitors in biochar, thereby maintaining an extremely high bacterial density over a long period. This provides a reasonable explanation for why BCB treatment still retained growth-promoting activity at 30 days in the present study (Nakahara et al. 2025). The carrier effect of biochar goes far beyond physical protection. By optimizing the microecology, it significantly improves the survival rate and persistence of functional bacteria. Consequently, BCB treatment significantly increased the root length and root number of *I. pes-caprae* by 101% and 63%, respectively, and was significantly superior to the single treatments. The same synergistic effect has been reported in studies using bacteria-derived biochar for the remediation of contaminated soil, where biochar as a microbial carrier exerted a more long-lasting biological effect (Cheng et al. 2025). The synergistic growth-promoting effect of the BCB treatment was reflected in multiple aspects, which is consistent with previous findings in similar systems. Regarding root development, the BCB treatment resulted in a root length of 9.17 cm and a root number of 10.33 at 30 days, which were significantly increased by 101% and 63% compared with CK, and also significantly better than the BC and B treatments. This result is highly consistent with the study by Liu et al. on rice, who reported that the combined application of biochar and phosphate-solubilizing bacteria significantly increased total root length, root surface area, and root tip number, and increased available phosphorus content in the culture solution by 309% compared with the control (Liu et al. 2022), indicating that biochar as a carrier indirectly optimized root architecture by improving phosphorus availability in the rhizosphere. Tahir et al. (2022) also confirmed that biochar as a microbial carrier could increase plant dry weight by 62% under saline-alkali soil conditions. Although the nutrient contents in plant tissues were not directly determined in this study, the optimized root architecture (significant increases in root length and root number) generally implies a larger absorption surface area. Yang et al. (2022) also demonstrated that the application of microbial inoculants improved the bioavailability of phosphorus and potassium by altering the soil bacterial community composition (significantly increasing the relative abundance of *Bacillus* and *Paenibacillus*).

#### Synergistic activation of leaf antioxidant enzyme activities and alleviation of membrane lipid peroxidation

The enhancement of antioxidant enzyme activities in *I. pes-caprae* under BCB treatment indicated that *F. velutipes* residue-based biochar in combination with *B. megaterium* YZS-M06 effectively strengthened stress resistance in *I. pes-caprae*. Previous studies have demonstrated that the application of biochar-based *Bacillus subtilis* inoculants can increase plant SOD activity by up to 3.5-fold (This study showed a 2.23-fold increase compared with the control), indicating that synergistic interactions between biochar and beneficial microorganisms are capable of activating systemic resistance responses in plants. In this study, BCB treatment was shown to concurrently enhance the activities of multiple key antioxidant enzymes, including SOD, POD, and CAT, while markedly reducing MDA content, These results indicate that BCB treatment may effectively alleviate oxidative damage by activating the plant’s antioxidant defense system. Moreover, the observed alleviation of membrane lipid peroxidation damage may not only be attributed to elevated antioxidant enzyme activities but may also benefit from improvements in the rhizosphere micro-environment (Zhang et al. 2025). Synergistic interactions between biochar and microorganisms may mitigate environmental stressors that induce oxidative stress at the source by buffering fluctuations in environmental pH and adsorbing harmful substances (Zhao et al. 2020). Collectively, these processes demonstrate the dual enhancement effects of “induction” and “buffering” exerted by the biochar carrier and functional microorganisms in regulating plant physiological status.

#### Synergistic effects enhancing stress resistance

On the one hand, microbial metabolism itself may directly contribute to osmotic regulatory substances. For example, previous studies have confirmed that strains such as *B. megaterium* are capable of secreting low-molecular-weight organic acids (e.g., pyruvate and malate) as well as plant hormones (e.g., indole-3-acetic acid) during metabolic processes (Kang et al. 2023). These compounds function not only as intermediates in sugar metabolism but also as signaling molecules that stimulate plants to synthesize increased levels of soluble sugars (Glick 2012). On the other hand, synergistic interactions between biochar and functional bacteria substantially improve soil fertility. It has been demonstrated that biochar loaded with functional bacteria can effectively increase soil organic matter, alkali-hydrolyzable nitrogen, and AK contents, thereby establishing a robust material basis for enhanced carbon fixation and metabolic activity in plants (Warren and Butler 2024). As essential osmotic regulatory substances and energy sources, the pronounced accumulation of soluble sugars not only directly maintains cellular osmotic homeostasis but also supplies the necessary substrates for extensive biosynthesis of the aforementioned antioxidant enzymes (Bolouri-Moghaddam et al. 2010). Regulation at the metabolic level, together with reinforcement of the antioxidant system, collectively constitutes the physiological foundation through which BCB treatment enhances the stress resistance of *I. pes-caprae* and supports sustained superior performance.

## Conclusion

This study reveals the synergistic mechanism of *F. velutipes* residue-derived biochar loaded with *B. megaterium* YZS-M06 in enhancing the growth and stress resistance of the coastal plant *I. pes-caprae*. It innovatively integrates the resource utilization of agricultural waste with microbial soil improvement technology, and constructs a composite material with both ecological restoration and plant growth-promoting functions. The growth of *I. pes-caprae* was significantly promoted by *F. velutipes* residue-based biochar loaded with *B. megaterium* YZS-M06 through synergistic interactions between biochar and functional bacteria. This synergy was reflected in the provision of a stable survival microenvironment for *B. megaterium* by biochar, while *B. megaterium* YZS-M06 facilitated the growth of *I. pes-caprae* via optimization of soil micro-ecology. With respect to stress resistance, BCB treatment activated the leaf antioxidant enzyme system and alleviated membrane lipid peroxidation damage, while simultaneously promoting the accumulation of osmotic adjustment substances through metabolic regulation. Additionally, BCB treatment drove functional optimization of rhizosphere microbial communities by regulating bacterial community structure, reshaping microbial network relationships, and optimizing key ecological functional pathways. Collectively, these effects elucidate the physiological and ecological mechanisms underlying the superiority of BCB treatment over single treatments in promoting plant growth and enhancing stress resistance. In future research, based on the findings of this study, we will conduct field control experiments and establish long-term monitoring plots to further verify the universality of these conclusions in complex coastal environments, we will also address the current data gap by including soil organic matter measurements in future investigations, so as to provide theoretical basis and technical support for the development of green remediation materials derived from agricultural and forestry wastes combined with functional microorganisms.

## Electronic Supplementary Material

Below is the link to the electronic supplementary material.

Supplementary Material 1

## Data Availability

Raw sequence data have been deposited in the National Genomics Data Center (NGDC) under accession number CRA035806.
