# Supplementary material for: Enoki mushroom residue-based biochar loaded with Bacillus megaterium YZS-M06 improves coastal sandy soil and promotes Ipomoea pes-caprae growth
Source: Bioresour Bioprocess. 2026 May 26;13(1):76. doi: 10.1186/s40643-026-01073-w (PMC13212864; doi:10.1186/s40643-026-01073-w)
Supplement: Supplementary file 1 — Supplementary Material 1 [file 40643_2026_1073_MOESM1_ESM.docx]

Enoki mushroom residue-based biochar loaded with *Bacillus megaterium* YZS-M06 improves coastal sandy soil and promotes *Ipomoea pes-caprae* growth

**Supplementary data**

Table S1 Soil chemical property indicators

| group | TK/(g/kg) | TN/(g/kg) | TP/(mg/kg) | NH_4_^+^-N(mg/kg) | NO_3_^-^-N(mg/kg) | AP(mg/kg) | AK(mg/kg) | AS(mg/kg) |
| --- | --- | --- | --- | --- | --- | --- | --- | --- |
| CK_1 | 4.35±0.26a | 0.10±0.02c | 49.93±8.25b | 0.13±0.01d | 0.09±0.00b | 8.19±0.23c | 21.33±0.15c | 17.46±1.1c |
| BC_1 | 4.60±0.73a | 0.15±0.05c | 79.19±4.09b | 0.34±0.03c | 0.06±0.01b | 8.71±0.07b | 21.47±0.48c | 26.39±2.68bc |
| B_1 | 4.59±0.70a | 0.26±0.02b | 160.19±4.23a | 0.43±0.01b | 0.16±0.01a | 8.75±0.05b | 45.58±0.27b | 35.67±1.82b |
| BCB_1 | 5.21±0.45a | 0.43±0.02a | 162.33±3.64a | 0.60±0.02a | 0.24±0.00a | 9.83±0.06a | 50.42±0.57a | 42.94±2.18a |
| CK_2 | 5.20±0.23c | 0.20±0.00d | 56.60±4.51c | 0.20±0.11c | 0.05±0.00b | 8.69±0.05b | 23.77±1.21c | 12.94±1.61c |
| BC_2 | 5.41±0.48c | 0.44±0.05c | 59.64±3.54c | 0.28±0.10bc | 0.06±0.02b | 9.24±0.44a | 24.31±0.88c | 15.12±2.06b |
| B_2 | 6.58±0.73b | 0.57±0.03b | 63.35±20.43b | 0.37±0.09ab | 0.07±0.00b | 9.29±0.17a | 47.06±0.61b | 26.51±1.43a |
| BCB_2 | 12.89±0.73a | 0.65±0.02a | 124.91±4.71a | 0.43±0.07a | 0.15±0.04a | 9.52±0.17a | 81.75±0.82a | 29.96±2.40a |

Note: Different lowercase letters indicate significant differences among the same indicator groups (*p* < 0.05).

Table S2 Soil enzyme activity indicators

| group | S-UE/(U/g) | S-ACP/(U/g) | S-LAP/(U/g) | S-SC/(U/g) |
| --- | --- | --- | --- | --- |
| CK_1 | 17.71±0.31d | 1934.74±151.99c | 0.23±0.02c | 5.05±0.05b |
| BC_1 | 24.38±1.13c | 1831.7±206.06c | 0.26±0.06b | 5.58±0.18b |
| B_1 | 38.23±0.19b | 2575.9±180.61b | 0.27±0.24b | 5.92±0.11b |
| BCB_1 | 58.13±0.4a | 4235.48±175.71a | 0.42±0.06a | 12.8±0.32a |
| CK_2 | 13.2±0.92d | 1597.21±233.33d | 0.52±0.04d | 0.57±0.07c |
| BC_2 | 19.29±2.72c | 2293.87±314.93c | 0.68±0.06c | 0.66±0.13c |
| B_2 | 35.06±0.62b | 2805.44±127.58b | 0.82±0.03b | 2.39±0.37b |
| BCB_2 | 42.39±0.42a | 4949.05±138.79a | 1.32±0.06a | 6.36±0.37a |

Note: Different lowercase letters indicate significant differences among the same indicator groups (*p* < 0.05).

Table S3 Sample alpha diversity index

| group | Richness | Shannon | Pielou | Chao1 |
| --- | --- | --- | --- | --- |
| CK_1 | 2266.67±46.81 | 7.93±0.1 | 0.71±0.01 | 2759.62±10.52 |
| BC_1 | 2018.33±36.26 | 7.91±0.04 | 0.72±0 | 2516.98±79.78 |
| B_1 | 1874.67±66.56 | 7.07±0.04 | 0.65±0 | 2415.85±140.89 |
| BCB_1 | 1527.33±30.07 | 5.76±0.23 | 0.55±0.02 | 1946.95±18.24 |
| CK_2 | 2253.67±152.77 | 8.24±0.22 | 0.74±0.01 | 2697.64±120.87 |
| BC_2 | 2264.33±98.80 | 8.24±0.21 | 0.74±0.01 | 2750.30±100.52 |
| B_2 | 2132.00±41.74 | 7.65±0.06 | 0.69±0.00 | 2670.99±22.12 |
| BCB_2 | 1781.33±15.86 | 6.04±0.12 | 0.56±0.01 | 2206.2±45.48 |

Note: Different lowercase letters indicate significant differences among the same indicator groups (*p* < 0.05).

Table S4. Relative abundance at the bacterial phylum level

| phylum | CK_1 | BC_1 | B_1 | BCB_1 | CK_2 | BC_2 | B_2 | BCB_2 |
| --- | --- | --- | --- | --- | --- | --- | --- | --- |
| Proteobacteria | 0.42±0.04ab | 0.46±0.04a | 0.32±0.01c | 0.26±0.05d | 0.4±0.05abc | 0.45±0.04a | 0.37±0.01bc | 0.22±0.02d |
| Firmicutes | 0.06±0.01d | 0.03±0.00d | 0.33±0.01b | 0.43±0.13a | 0.08±0.03d | 0.03±0.00d | 0.22±0.03c | 0.50±0.02a |
| Actinobacteriota | 0.16±0.01a | 0.13±0.02b | 0.08±0.01c | 0.05±0.02d | 0.13±0.02ab | 0.14±0.01ab | 0.13±0.01ab | 0.05±0.01d |
| Bacteroidota | 0.06±0bcd | 0.13±0.02a | 0.08±0.00bc | 0.07±0.02bcd | 0.05±0.01cd | 0.08±0.01b | 0.07±0.01bcd | 0.04±0.00d |
| Cyanobacteria | 0.09±0.03a | 0.02±0.01a | 0.09±0.01a | 0.12±0.17a | 0.08±0.12a | 0.04±0.04a | 0.04±0.01a | 0.07±0.02a |
| Acidobacteriota | 0.05±0.01a | 0.06±0.02a | 0.02±0.00b | 0.02±0.01b | 0.05±0.01a | 0.06±0.01a | 0.04±0.01ab | 0.05±0.01a |
| Chloroflexi | 0.04±0.01ab | 0.02±0.01cd | 0.02±0.00de | 0.01±0.00e | 0.05±0.01a | 0.05±0.01a | 0.03±0.00bc | 0.02±0.00cde |
| Gemmatimonadota | 0.03±0.00b | 0.05±0.01a | 0.02±0.00c | 0.01±0.00c | 0.05±0.01a | 0.05±0.01a | 0.03±0.00b | 0.01±0.00c |
| Bdellovibrionota | 0.04±0.00a | 0.03±0.01ab | 0.02±0.00c | 0.01±0.00d | 0.03±0.00ab | 0.03±0.00ab | 0.03±0.00b | 0.01±0.00d |
| Patescibacteria | 0.02±0.00b | 0.01±0.00b | 0.01±0.00b | 0.00±0.00b | 0.04±0.01a | 0.02±0.00b | 0.02±0.01b | 0.00±0.00b |
| others | 0.04±0.00a | 0.05±0.01a | 0.02±0.00bc | 0.02±0.00c | 0.03±0.01ab | 0.04±0.01a | 0.04±0.01ab | 0.02±0.00bc |

Note: Different lowercase letters indicate significant differences among the same indicator groups (*p* < 0.05).

Table S5. Relative abundance at the bacterial genus level

| genus | CK_1 | BC_1 | B_1 | BCB_1 | CK_2 | BC_2 | B_2 | BCB_2 |
| --- | --- | --- | --- | --- | --- | --- | --- | --- |
| *Bacillus* | 0.05±0.01d | 0.04±0.00d | 0.27±0.01c | 0.47±0.13b | 0.08±0.04d | 0.03±0.01d | 0.20±0.03c | 0.58±0.03a |
| *Sphingomonas* | 0.13±0.02a | 0.09±0.00bc | 0.08±0.00cd | 0.05±0.01e | 0.14±0.01a | 0.13±0.00a | 0.11±0.01b | 0.07±0.00de |
| *Lysobacter* | 0.08±0.01b | 0.12±0.01a | 0.06±0.00c | 0.03±0.01d | 0.07±0.01c | 0.08±0.01b | 0.06±0.01c | 0.02±0.00d |
| *Arthrobacter* | 0.09±0.01a | 0.08±0.03ab | 0.04±0.00c | 0.03±0.02c | 0.06±0.01bc | 0.09±0.02ab | 0.07±0.01abc | 0.03±0.00c |
| *Microvirga* | 0.02±0.00d | 0.03±0.01cd | 0.05±0.00ab | 0.04±0.01b | 0.03±0.01cd | 0.02±0.00d | 0.06±0.00a | 0.04±0.01bc |
| *Ramlibacter* | 0.04±0.01b | 0.05±0.00a | 0.02±0.00d | 0.01±0.00e | 0.04±0.00b | 0.05±0.00ab | 0.03±0.00c | 0.01±0.00e |
| *Flavisolibacter* | 0.02±0.00cd | 0.05±0.01a | 0.04±0.00b | 0.03±0.01bcd | 0.02±0.00d | 0.03±0.00bc | 0.03±0.01bcd | 0.02±0.00d |
| *Massilia* | 0.07±0.05a | 0.05±0.01a | 0.01±0.00a | 0.01±0.00a | 0.03±0.00a | 0.03±0.00a | 0.02±0.00a | 0.01±0.00a |
| *Leptolyngbya_EcFYyyy-00* | 0.02±0.00a | 0.00±0.00a | 0.04±0.01a | 0.10±0.17a | 0.02±0.04a | 0.00±0.00a | 0.00±0.00a | 0.01±0.00a |
| *Ensifer* | 0.01±0.00b | 0.02±0.00ab | 0.02±0.00ab | 0.02±0.01ab | 0.02±0.01ab | 0.02±0.01ab | 0.03±0.00a | 0.02±0.00ab |
| others | 0.46±0.02a | 0.48±0.00a | 0.38±0.02b | 0.22±0.06c | 0.51±0.03a | 0.51±0.02a | 0.40±0.01b | 0.21±0.02c |

Note: Different lowercase letters indicate significant differences among the same indicator groups (*p* < 0.05).

Table S6 Sample network topology index

| Group | CK_1 | BCB_1 | B_1 | BC_1 | CK_2 | BCB_2 | B_2 | BC_2 |
| --- | --- | --- | --- | --- | --- | --- | --- | --- |
| num edges(L) | 6119 | 6270 | 5977 | 6060 | 5968 | 5772 | 6449 | 6291 |
| num pos edges | 3448 | 4000 | 3013 | 3009 | 3551 | 3059 | 3254 | 3202 |
| num neg edges | 2671 | 2270 | 2964 | 3051 | 2417 | 2713 | 3195 | 3089 |
| centralization degree | 0.05 | 0.08 | 0.05 | 0.02 | 0.06 | 0.08 | 0.07 | 0.06 |
| relative modularity | 8.62 | 8.77 | 9.47 | 9.65 | 7.91 | 9.18 | 9.64 | 8.44 |

Table S7 Sample growth indicators

| group | Bud number | Bud length/cm | Plant height/cm | Root number | Root length/cm | Fresh weight/g | Dry weight/g |
| --- | --- | --- | --- | --- | --- | --- | --- |
| CK_1 | 3.00±0.00b | 1.73±0.49c | 6.93±0.06d | 3.33±0.53a | 4.27±0.21d | 2.11±0.10c | 3.11±0.14c |
| BC_1 | 5.33±1.15a | 3.43±0.49b | 7.33±0.15c | 4.33±0.08a | 5.30±0.26c | 2.53±0.11bc | 3.95±0.49bc |
| B_1 | 5.67±1.15a | 3.90±0.17b | 8.43±0.12b | 6.33±0.58a | 5.73±0.06b | 2.87±0.35ab | 4.94±0.17b |
| BCB_1 | 7.00±0.00a | 6.47±0.15a | 9.07±0.12a | 6.67±0.53a | 6.17±0.29a | 3.15±0.31a | 6.91±0.79a |
| CK_2 | 6.33±0.58b | 4.47±0.06b | 7.67±0.47a | 6.33±0.15c | 4.57±0.40b | 0.33±0.01c | 0.54±0.05c |
| BC_2 | 8.00±0.00ab | 4.60±0.46b | 9.3±0.87a | 8.00±0.00b | 6.03±0.35b | 0.37±0.01b | 0.59±0.01bc |
| B_2 | 10.00±1.15a | 5.63±0.35b | 9.67±0.61a | 9.00±0.00b | 7.33±0.53ab | 0.44±0.00a | 0.64±0.04b |
| BCB_2 | 10.67±1.15a | 7.77±0.93a | 9.83±1.19a | 10.33±0.58a | 9.17±0.76a | 0.46±0.01a | 0.92±0.02a |

Note: Different lowercase letters indicate significant differences among the same indicator groups (*p* < 0.05).

Table S8 Enzyme activity indicators of sample leaves

| group | CAT/(U/g) | POD/(U/g) | SOD/(U/g) | MDA/(U/g) | Soluble suger/(mg/g) |
| --- | --- | --- | --- | --- | --- |
| CK_1 | 95.58±0.03c | 18122.65±757.05c | 75.47±5.13c | 25.66±0.39a | 11.1±0.04d |
| BC_1 | 97.30±0.01b | 22635.55±1158.85b | 95.57±1.78b | 22.47±0.33b | 11.71±0.09c |
| B_1 | 98.69±0.01a | 24051.65±1423.45ab | 148.98±24.28a | 18.39±0.09c | 12.65±0.11b |
| BCB_1 | 99.02±0.04a | 25656.40±1421.00a | 154.63±5.18a | 4.65±0.16d | 13.35±0.12a |
| CK_2 | 140.01±0.15d | 11638.68±144.14a | 220.42±15.61a | 22.19±0.14a | 12.1±0.05d |
| BC_2 | 140.33±0.19c | 28365.56±217.92b | 427.46±16.81a | 18.02±0.28b | 15.62±0.04c |
| B_2 | 146.53±0.29b | 38446.87±427.98c | 483.09±22.43a | 16.79±0.30c | 17.72±0.12b |
| BCB_2 | 149.88±0.21a | 40420.33±516.01d | 490.5±19.14a | 12.97±0.28d | 37.31±0.18a |

Note: Different lowercase letters indicate significant differences among the same indicator groups (*p* < 0.05).


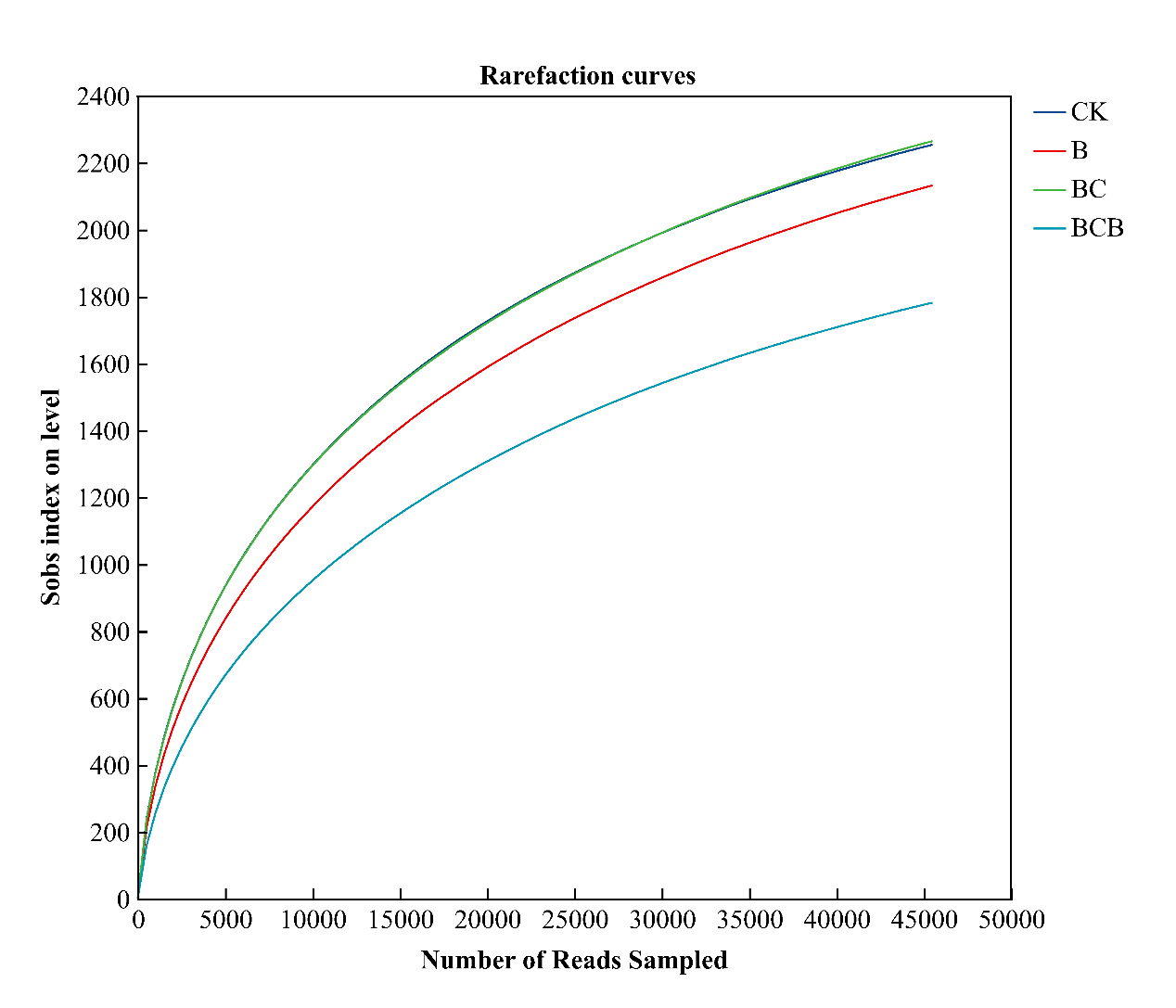


Figure S1 Sample dilution curve
